# Deterministic Light-to-Voltage Conversion with a Tunable Two-Dimensional Diode

**DOI:** 10.1021/acsphotonics.2c00727

**Published:** 2022-07-21

**Authors:** Mingde Du, Xiaoqi Cui, Bin Zhang, Zhipei Sun

**Affiliations:** † Department of Electronics and Nanoengineering, Aalto University, Espoo FI-02150, Finland; ‡ QTF Centre of Excellence, Department of Applied Physics, Aalto University, Espoo FI-00076, Finland; § Key Laboratory of In-Fiber Integrated Optics of Ministry of Education, College of Physics and Optoelectronic Engineering, Harbin Engineering University, Harbin 150001, China

**Keywords:** two-dimensional heterostructure, photovoltaic effect, photoconduction effect, indium selenide, two-dimensional metallic materials, dielectric screening, photocurrent mapping

## Abstract

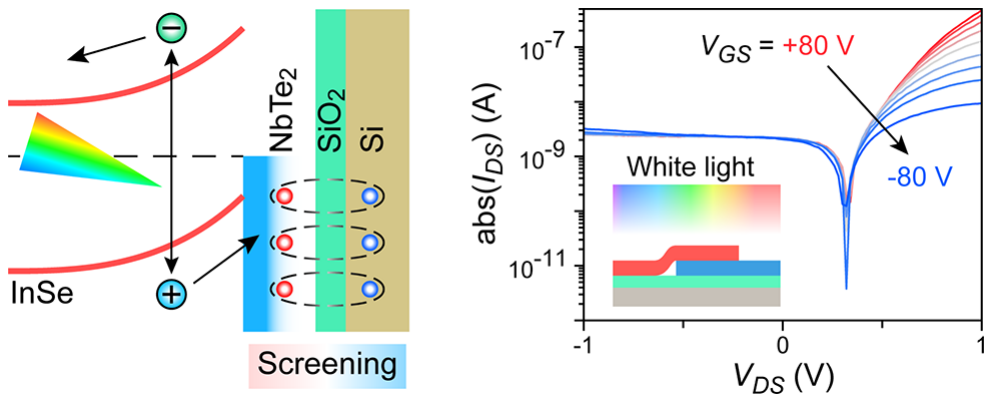

Heterojunctions accompanied by energy barriers are of significant importance in two-dimensional materials-based electronics and optoelectronics. They provide more functional device performance, compared with their counterparts with uniform channels. Multimodal optoelectronic devices could be accomplished by elaborately designing band diagrams and architectures of the two-dimensional junctions. Here, we demonstrate deterministic light-to-voltage conversion based on strong dielectric screening effect in a tunable two-dimensional Schottky diode based on semiconductor/metal heterostructure, where the resultant photovoltage is dependent on the intensity of light input but independent of gate voltage. The converted photovoltage across the diode is independent of gate voltage under both monochromatic laser and white light illumination. In addition, the Fermi level of two-dimensional semiconductor area on dielectric SiO_2_ is highly gate-dependent, leading to the tunable rectifying effect of this heterostructure, which corporates a vertical Schottky junction and a lateral homojunction. As a result, a constant open-circuit voltage of ∼0.44 V and a hybrid “photovoltaic + photoconduction” photoresponse behavior are observed under 1 μW illumination of 403 nm laser, in addition to an electrical rectification ratio up to nearly 10^4^. The scanning photocurrent mappings under different bias voltages indicate that the switchable operation mode (photovoltaic, photoconduction, or hybrid) depends on the bias-dependent effective energy barrier at the two-dimensional semiconductor–metal interface. This approach provides a facile and reliable solution for deterministic on-chip light-to-voltage conversion and optical-to-electrical interconnects.

## Introduction

Two-dimensional (2D) semiconductors have been extensively studied in the past two decades, especially for electronics and optoelectronics.^1−6^ The alignment between band gaps of different 2D semiconductors varies a lot,^7,8^ thus heterojunctions with effective rectifying effect and photovoltaic effect could be readily built at the interfaces between different 2D semiconductors.^9,10^ However, the construction of heterojunctions increases the complexity of the mass production of functional devices, and the inherent relatively high energy barriers at the interfaces limit the electrical conduction of device channels. An alternative to achieve ideal junctions without sacrificing electrical conduction is a homojunction formed with areas of the same material but in different states (doping, dielectric environment, etc.). For a certain 2D semiconductor material, the size and transition type (direct or indirect) of its band gap are highly dependent on its thickness,^11−13^ giving us opportunities for building homojunctions with the same material of different thicknesses.^14^ The 2D homojunctions based on thickness tuning demonstrate a weaker rectifying effect than heterojunctions, as the energy barrier formed at the interface of different thicknesses is finite, and the width of depletion region is always as short as nanometer scale.^15,16^ To increase the energy band mismatch in 2D homojunctions and enhance rectifying effect, the uniform 2D semiconductors are locally doped through gate voltage or chemical doping or placed on ununiform dielectric substrates.^17−21^ Another approach to locally modulate the doping level of a 2D semiconductor is charge redistribution, which happens when a 2D semiconductor contacts with a metallic material.^22,23^

Two-dimensional metallic materials have been successfully synthesized by chemical methods and employed to lower the Schottky barrier height (SBH) at the contact interfaces between 2D semiconductors and metallic electrodes.^24−29^ In contrast, SBH at the contact interfaces formed with conventional metals (e.g., Au, Ni, Pt) that are deposited by evaporation and result in metal-induced gap states (MIGS) is always quite high.^8,30,31^ In addition to facilitating charge carrier transport, the 2D metal–semiconductor interfaces could also induce Schottky junctions through charge redistribution,^22,32^ demonstrating a high rectification ratio or photovoltaic effect with an external quantum efficiency (EQE) up to 55%.^32,33^ The previously published works of 2D metal–semiconductor interfaces mostly involve lateral junctions at atomic scale or vertical junctions formed with ultrathin flakes.^25−29^ Therefore, the electrical performance is highly tunable with gate voltage.^34^ In a published computational work, the authors show that the stacking sequence of a vertical 2D graphene–MoS_2_ junction could significantly affect the electrical transport behavior^35^ because the stacking sequence determines electronic density distribution in the device channel under a gate voltage. Furthermore, the thickness of graphene in a graphene–silicon heterojunction could heavily affect the tuning efficiency of charge density of silicon with gate voltage,^36^ and ultimately, the graphene layer could almost totally screen the gating effect when it is thicker than 10 layers. Accordingly, the doping level of a 2D semiconductor could be locally modulated by charge redistribution when it interfaces with a 2D metallic material, and the formed energy barrier would be independent of gate voltage if the 2D metallic material is thick enough.

Here, gate-independent light-to-voltage conversion is achieved with a 2D semiconductor/metal heterostructure, demonstrating a gate-tunable rectifying effect. The deliberately stacked 2D semiconductor/metal heterostructure actually incorporates a Schottky junction and a homojunction. First, the results of gate voltage-dependent output *I–V* curves measurements and scanning photocurrent mappings (SPMs) indicate gate-independent photovoltage generation, resulting from effective dielectric screening at the 2D metal–semiconductor interface. Second, a highly gate-tunable diode is revealed with *I–V* curves, indicating that a homojunction diode is successfully built with the 2D semiconductor that is locally doped through charge redistribution after contacting the 2D metallic material. Finally, the bias-dependent transition of the photoresponse mechanism is illustrated with SPMs.

## Results and Discussion

**Figure 1:**
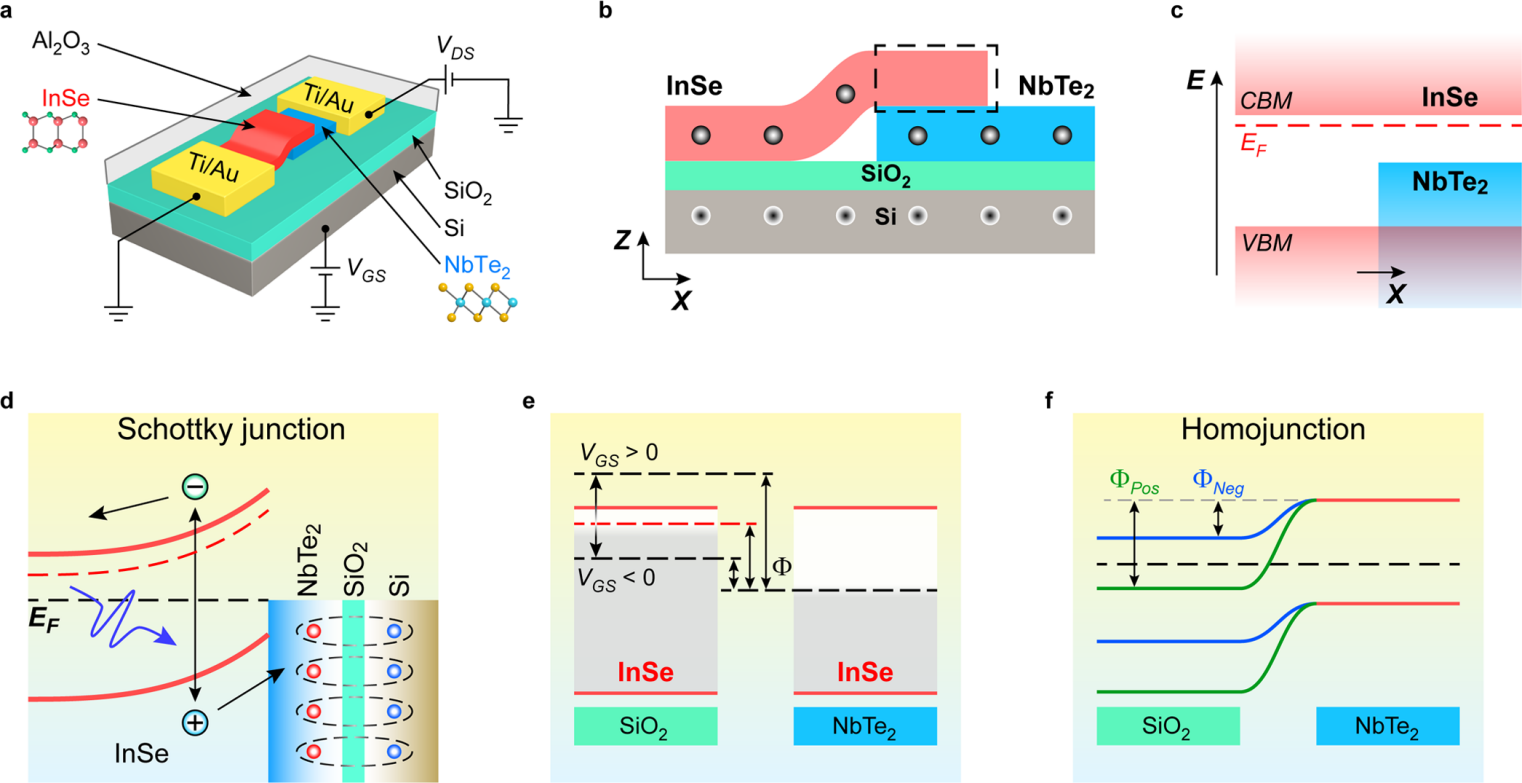
Device structure and band diagram. (a) Schematic of the InSe/NbTe_2_ heterostructure device. Metallic NbTe_2_ is placed on the bottom and overlaps with part of InSe, and an Al_2_O_3_ layer is deposited for protection. *V*_GS_: gate voltage. *V*_DS_: drain-source bias voltage. (b) Cross-sectional schematic of InSe/NbTe_2_ heterostructure. The circles with gradient colors indicate the opposite charges induced by *V*_GS_ in 2D materials and the silicon substrate. Because of dielectric screening, the InSe area (dashed box) on NbTe_2_ is not doped by *V*_GS_. (c) Relative band alignment between InSe and NbTe_2_. CBM: conduction band minimum. VBM: valence band maximum. The dashed line indicates the intrinsic Fermi level (*E*_F_) of InSe. (d) Band diagram at the interface between InSe and NbTe_2_. Red and black dashed lines indicate the Fermi level of InSe before and after contact. Photoexcited electron–hole pairs are separated by the built-in electric field. *V*_GS_ is assumed to have no effect on this Schottky junction, as the field effect is totally screened by the mobile charges in metallic NbTe_2_. (e) Fermi-level mismatch Φ between the InSe areas on SiO_2_ and NbTe_2_ at different *V*_GS_. (f) Band diagram of the homojunction formed between InSe areas on SiO_2_ and NbTe_2_. The height of the energy barrier in this homojunction is increased when *V*_GS_ is switched from negative (Φ_Neg_) to positive (Φ_Pos_).

In this study, the device is fabricated with mechanically exfoliated 2D flakes. Briefly, metallic 1T-phase NbTe_2_ and semiconducting InSe are exfoliated, and thick flakes are transferred to a substrate of p-doped silicon covered with 300 nm thick SiO_2_. Thus, a heterostructure with NbTe_2_ on the bottom is formed (Figure 1a).^37^ Followed by patterning and depositing Ti/Au electrodes, an Al_2_O_3_ layer is deposited to prevent the whole device from being damaged in the air, and potentially induces n-doping to the InSe flake.^38^ As illustrated in the schematic of Figure 1a, the Ti/Au electrode deposited on InSe is grounded, and drain-source bias voltage *V*_DS_ and gate voltage *V*_GS_ are applied at the Ti/Au electrode on NbTe_2_ and the conductive silicon substrate on the back side, respectively. In the SPMs measurements, a 403 or 532 nm laser beam with a power of ∼1 μW is illuminated through a 20× objective above the device and scanned in the whole device area. An optical microscope image of the device is shown in Figure S1. The 2D materials involved in the device are identified with Raman and photoluminescence spectra, and the results are demonstrated in Figure S2. A cross-sectional schematic of the device is shown in Figure 1b, and the thicknesses of InSe and NbTe_2_ are ∼31.75 and ∼33.43 nm based on the measurement with an atomic force microscope (AFM, Figure S3). The special stacking sequence leads to strong electrostatic screening from thick metallic NbTe_2_ at the overlapping area,^35,36^ but it is rarely observed in extremely thin 2D devices.^33,39^ As a result, the InSe area on SiO_2_ is effectively doped by the charges (circles in Figure 1b) accumulated by *V*_GS_ applied via conductive Si substrate, whereas the InSe area on metallic NbTe_2_ (dashed box in Figure 1b) is independent of *V*_GS_ because the gating effect is screened by metallic NbTe_2_. The band alignment between InSe and NbTe_2_ is shown in Figure 1c. The Fermi level of n-doped InSe is higher than that of metallic NbTe_2_. Consequently, charge redistribution and band bending happen at the InSe/NbTe_2_ overlapping area after contact, leading to the formation of a Schottky junction (Figure 1d). A built-in electric field is formed in the Schottky junction. Thus, the photoexcited electron–hole pairs are efficiently separated at the interface. The separated electrons and holes drift to InSe and NbTe_2_, respectively. Due to dielectric screening, the built-in electric field and photocarriers separation are almost independent of *V*_GS_. In addition, the Fermi level (black dashed line in Figure 1d) of the InSe area on NbTe_2_ is lowered compared with its intrinsic Fermi level (red dashed line in Figure 1d) because of charge redistribution, and a significant Fermi-level mismatch (Φ in Figure 1e) is induced between the InSe areas on SiO_2_ and NbTe_2_ after stacking. This Fermi-level mismatch is readily tunable with *V*_GS_, which only tunes the Fermi level of InSe on SiO_2_. Based on this Fermi-level mismatch, a homojunction is assumed to form between the InSe areas on SiO_2_ and NbTe_2_ (Figure 1f). Since the Fermi-level mismatch is dependent on *V*_GS_, the energy barrier in this homojunction is tunable with *V*_GS_. Overall, this heterostructure incorporates a gate-independent InSe/NbTe_2_ Schottky junction and a gate-tunable InSe homojunction.

**Figure 2:**
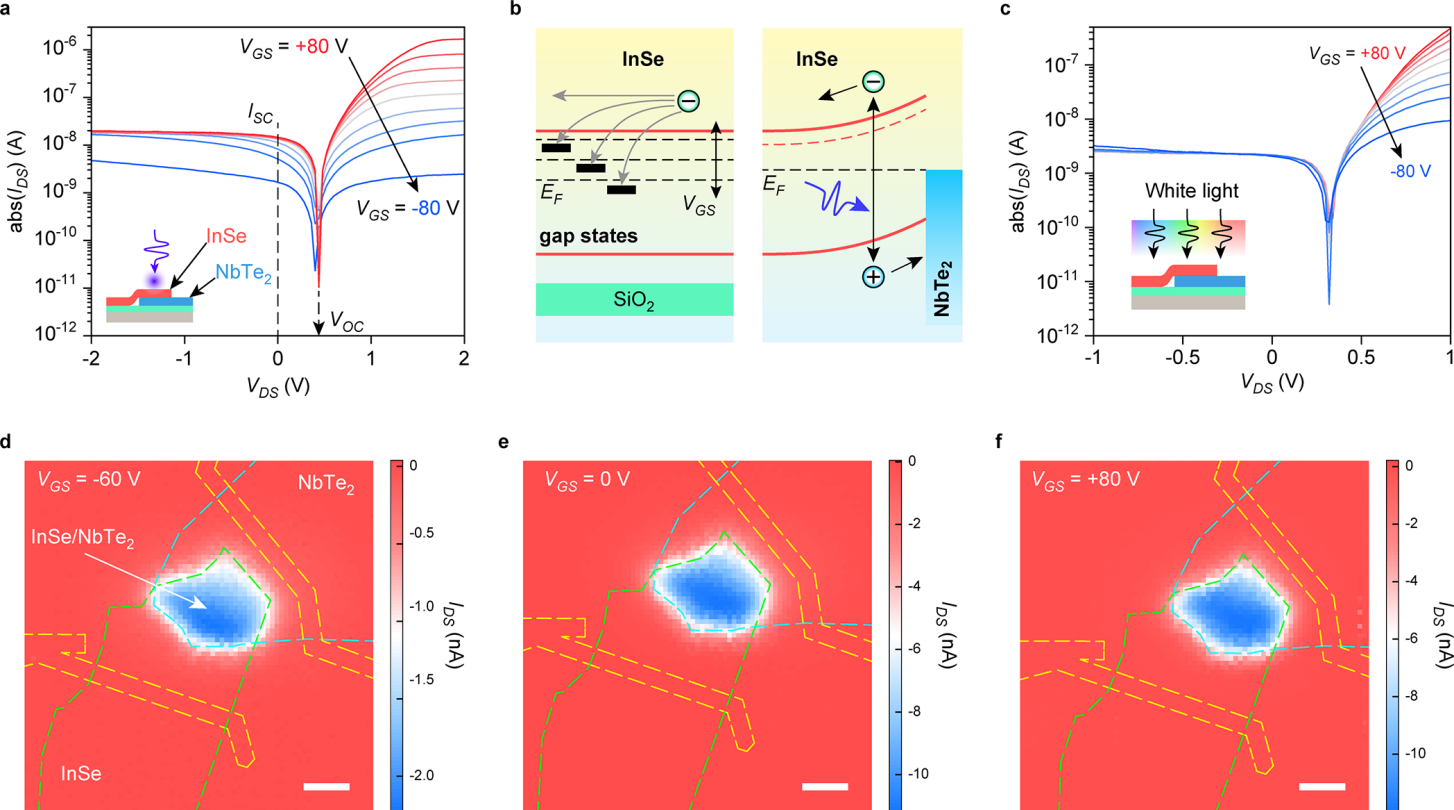
Gate-independent light-to-voltage conversion. (a) Output *I*_DS_–*V*_DS_ curves measured with the 403 nm laser spot of 1 μW focusing at InSe/NbTe_2_ heterostructure (inset). *I*_SC_: short-circuit current. *V*_OC_: open-circuit voltage. (b) Effect of *V*_GS_ on *I*_SC_. Only the gap states (black bars) above the Fermi level (*E*_F_) of InSe, which is highly tunable with *V*_GS_ (dashed lines in the left panel), could trap (gray arrows) photocarriers and lower the *I*_SC_. (c) Output *I*_DS_–*V*_DS_ curves measured at different *V*_GS_ under white light illumination (inset). (d–f) Scanning photocurrent mappings measured with 532 nm laser illumination of 1 μW and *V*_DS_ = 0, when different gate voltages of *V*_GS_ = −60 V (d), 0 V (e), and +80 V (f) are applied. Green, blue, and orange dashed lines outline the positions of InSe, NbTe_2_, and Ti/Au electrodes, respectively. Scale bars, 5 μm.

To verify the dielectric screening effect illustrated in Figure 1b,d, output *I*_DS_–*V*_DS_ curves are measured with the focused 403 nm laser spot of 1 μW illuminating the InSe/NbTe_2_ heterostructure area and are depicted with the absolute value of *I*_DS_ (abs(*I*_DS_)) shown in log-scale in Figure 2a. These results are in stark contrast to the previously published works,^10,40−42^ where both short-circuit current (*I*_SC_) and open-circuit voltage (*V*_OC_) are highly dependent on *V*_GS_, suggesting a unique photoresponse mechanism. *V*_OC_ is almost fixed at 0.44 V and independent of *V*_GS_, while the *I*_SC_ is significantly increased when *V*_GS_ ranges from −80 to 0 V and almost saturates at positive *V*_GS_. The *V*_GS_-independent *V*_OC_ is quite valuable for the application of on-chip light-to-voltage conversion,^43^ where a stable photovoltage is desired under a certain light illumination condition. The photovoltaic effect could be optimized by carefully selecting a 2D metal with proper work function.^44^ The gate-dependent *I*_SC_ (Figure 2a) could be explained with the band diagram in Figure 2b, indicating that the excited electrons generated by the photovoltaic effect in the vertical InSe/NbTe_2_ Schottky junction (Figure 1d) drift in horizontal direction to the InSe channel on SiO_2_. The drifted electrons have a shorter mean free path when negative *V*_GS_ is applied and the Fermi level of the InSe channel on SiO_2_ is pushed downward, as there are plenty of gap states (black bars in Figure 2b) over the Fermi level of InSe channel and many of the drifted electrons are trapped (gray arrows in Figure 2b) by the gap states. However, the trapping effect is significantly decreased, and *I*_SC_ is retained at a high *V*_GS_ when most of the gap states are filled with the accumulated charges. The assumption of gate-dependent *I*_SC_ could be confirmed with the photoresponse to wide-field white light (Figure 2c) that illuminates the whole device area, which is the common illumination condition in practical photodetection and photovoltaics applications. The *V*_GS_-independent *V*_OC_ is also verified with light illumination of different intensities (Figure S4). The major difference between focused laser illumination and white light illumination is the excitation of the InSe area on SiO_2_. Most of the gap states in the InSe area on SiO_2_ are filled because of the photogating effect under white light illumination, even if high negative *V*_GS_ is applied. It is reasonable to conclude that the *I*_SC_ is retained at a relatively high level when the InSe area on SiO_2_ is illuminated as well. Therefore, the low *I*_SC_ at negative *V*_GS_ under focused laser illumination results from the gap states, and an appreciable level of n-doping from the Al_2_O_3_ layer is beneficial for the photovoltaic effect in this device.^38^ The dielectric screening effect is further investigated with SPMs at *V*_DS_ = 0 measured under the illumination of a 532 nm laser. The results are demonstrated in Figure 2d–f. The mappings confirm the strong photovoltaic effect at the InSe/NbTe_2_ heterostructure, when the *V*_GS_ is widely changed from −60 to +80 V. All of the above results in Figure 2 indicate that the InSe/NbTe_2_ Schottky junction is gate-independent, as a result of strong dielectric screening effect. Thus, the photovoltage generated with this device is determined by the illumination condition and its inherent band alignment, and is highly resistant to electrical disturbance like gate voltage. The photovoltage generated under the illumination of modulated laser is shown in Figure S5. Based on the results, the response time is ∼0.33 ms, and this value can be further improved by optimizing the interfaces in the device.

**Figure 3:**
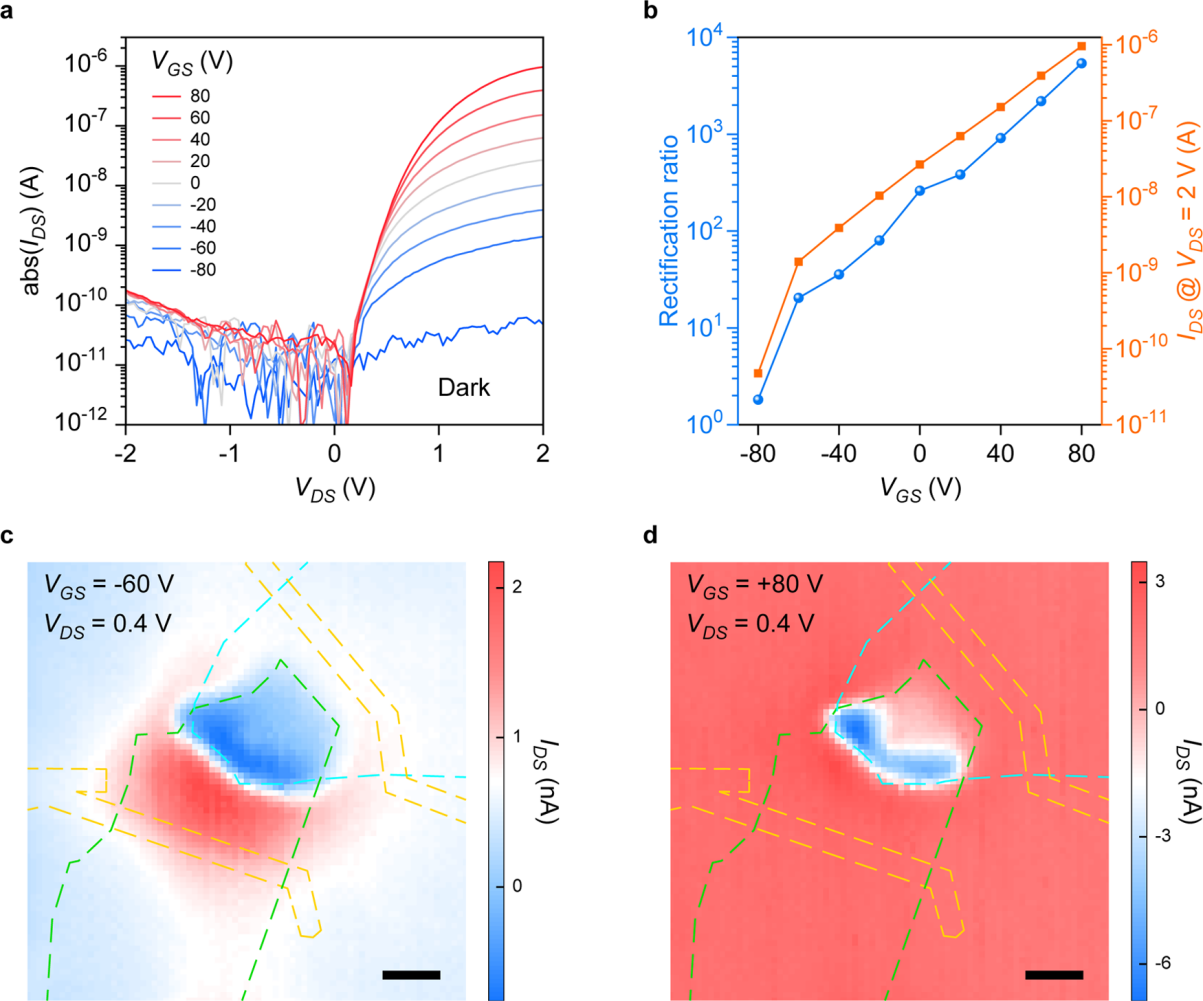
Gate tunability of the diode. (a) Gate-dependent *I*_DS_–*V*_DS_ curves measured under dark condition. (b) Gate-tunable rectification ratio and *I*_DS_ at *V*_DS_ = 2 V. (c, d) Scanning photocurrent mappings measured with 403 nm laser illumination of 1 μW and *V*_DS_ = 0.4 V, when gate voltages of *V*_GS_ = −60 V (c) and +80 V (d) are applied. Green, blue, and orange dashed lines outline the positions of InSe, NbTe_2_, and Ti/Au electrodes, respectively. Scale bars, 5 μm.

Subsequently, gate tunability of the heterostructure diode is investigated. The output *I*_DS_–*V*_DS_ curves measured in the dark condition (Figure 3a) are highly dependent on *V*_GS_, as assumed before. Based on these curves, this device is in reverse and forward bias at negative and positive *V*_DS_, respectively. This trend agrees well with the band diagram in Figure 1d,f, indicating that a negative *V*_DS_ applied on NbTe_2_ tends to increase the energy barrier height in both the InSe/NbTe_2_ Schottky junction and the InSe homojunction and switch the junctions to high resistance state, whereas a positive *V*_DS_ decreases the energy barrier height and switch the junctions to low resistance state. The measured *I*_DS_ at a reverse bias of *V*_DS_ = −1 V is lower than 10^–10^ A, which is an extremely low value compared with other published 2D diodes,^20,40^ indicating that effective energy barrier height and width are obtained in this heterostructure.^32^ Furthermore, the *I*_DS_ at positive *V*_DS_ is highly tunable with *V*_*GS*_, as indicated by the rectification ratio (absolute value of the ratio between *I*_DS_ at *V*_DS_ = +2 V and *V*_DS_ = −2 V) and *I*_DS_ at *V*_DS_ = +2 V demonstrated in Figure 3b. As the *V*_GS_ shifts from −80 to +80 V, the rectification ratio is incrementally increased to nearly 10^4^, and *I*_DS_ at *V*_DS_ = +2 V is amplified with a factor of >10^4^. As discussed in the above section, the effect of *V*_GS_ is effectively screened by NbTe_2_ (Figure 1b,d). Thus, the gate tunability of this device mainly arises from the InSe area on SiO_2_. As illustrated in Figure 1e,f, the Fermi level of the InSe area on SiO_2_ rises and the energy barrier height in InSe homojunction is increased, when *V*_GS_ changes from −80 to +80 V. Simultaneously, the conductance of the InSe area on SiO_2_ is increased by high *V*_GS_ that increases the density of mobile carriers in InSe. The synergistic modulation of energy barrier height and channel conductance leads to the modulation of on-state current in a wide range. The gate tunability is also investigated with SPM measurement at the same *V*_DS_ of 0.4 V but different *V*_GS_, as shown in Figure 3c,d. The significant negative photocurrent is obtained at the InSe/NbTe_2_ heterostructure under the two different *V*_GS_, indicating the robustness of photovoltaic effect in the InSe/NbTe_2_ Schottky junction. Nevertheless, notable photocurrent when the laser beam is focused at the InSe area on SiO_2_ is only obtained at *V*_GS_ = −60 V, while it is ignorable at *V*_GS_ = +80 V. Overall, the gate-tunable photoconduction effect at the InSe area on SiO_2_ competes with the gate-independent photovoltaic effect in InSe/NbTe_2_ Schottky junction, leading to the distinct SPM patterns in Figure 3c,d. Obviously, this device works in “photoconduction + photovoltaic” hybrid mode and photovoltaic mode at *V*_GS_ = −60 and +80 V, respectively. The dependence of photoresponse at the InSe area on SiO_2_ on *V*_GS_ could be explained with the band diagram in Figure 1f. The energy barrier height in InSe homojunction is decreased at negative *V*_GS_; thus, the photocarriers generated by the photoconduction effect in the InSe area on SiO_2_ could be extracted more efficiently, leading to a more significant photocurrent at *V*_GS_ = −60 V. Therefore, the InSe homojunction is highly gate-tunable in terms of rectification ratio, field effect current On/Off ratio, and photoresponse, in contrast to the gate-independent InSe/NbTe_2_ Schottky junction.

**Figure 4:**
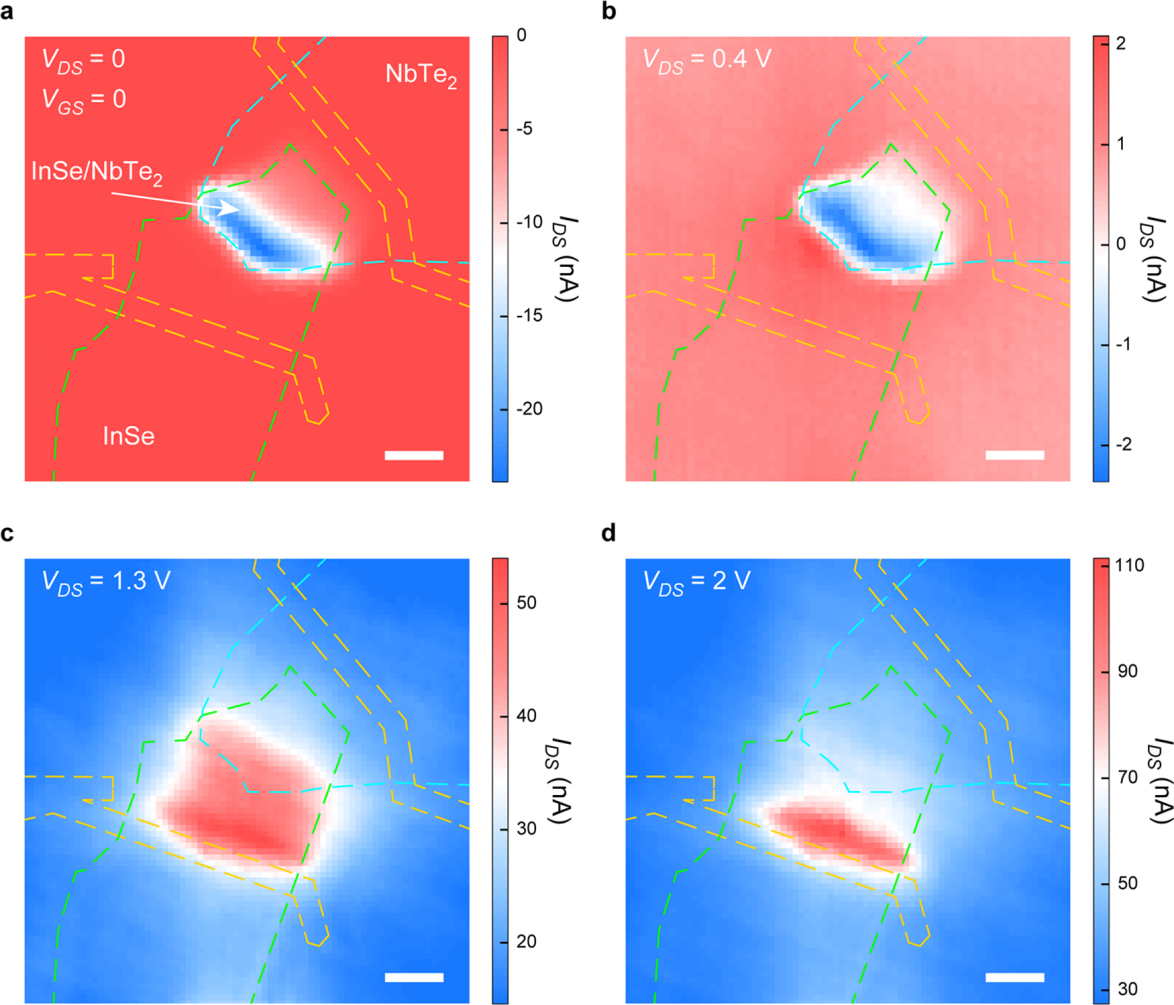
Bias-dependent transition of photoresponse mechanism. (a–d) Scanning photocurrent mappings measured with 403 nm laser illumination of 1 μW and *V*_GS_ = 0, when different bias voltages of *V*_DS_ = 0 V (a), 0.4 V (b), 1.3 V (c), and 2 V (d) are applied. Green, blue, and orange dashed lines outline the positions of InSe, NbTe_2_, and Ti/Au electrodes, respectively. Scale bars, 5 μm.

As illustrated in the band diagrams of Figure 1d,f, the height of energy barriers in the InSe/NbTe_2_ Schottky junction and InSe homojunction is tunable with *V*_DS_. To reveal the dependence of the photoresponse mechanism on *V*_DS_, SPMs at gate voltage *V*_GS_ = 0 are carried out and the results are demonstrated in Figure 4. At extreme bias voltage of *V*_DS_ = 0–2 V, significant photoresponse is only observed at InSe/NbTe_2_ overlapping area (photovoltaic effect, Figure 4a) and InSe area on SiO_2_ (photoconduction effect, Figure 4d), respectively. This dependence of photoresponse mechanism on *V*_DS_ is consistent with the previously published work.^37^ In another published work, photovoltaic and photogating effects are also observed in a homogeneous MoS_2_ transistor with asymmetric metal electrodes.^45^ Under a bias voltage of *V*_DS_ = 0.4 V, which is quite close to the *V*_OC_ = 0.44 V in Figure 2a, this device exhibits a hybrid photoresponse as shown in Figure 4b. In this case, positive and negative photocurrents are obtained when the laser beam illuminates the InSe area on SiO_2_ in proximity to NbTe_2_ and the InSe/NbTe_2_ overlapping area, respectively, indicating a hybrid photoresponse of coexisting photovoltaic and photoconduction effect at *V*_GS_ = 0 V and *V*_DS_ = 0.4 V. Once the bias voltage is much higher than *V*_OC_ = 0.44 V in Figure 2a, such as *V*_DS_ = 1.3 V in Figure 4c, positive photocurrent is observed in a broad InSe area on the substrate spanning SiO_2_ and NbTe_2_. Based on the band diagrams in Figure 1d,f, a substantially high positive *V*_DS_ applied via NbTe_2_ could “flatten” the band bending in both the Schottky junction and homojunction. Consequently, the photovoltaic effect in InSe/NbTe_2_ Schottky junction almost disappears, and the InSe area on NbTe_2_ responds to light illumination in a photoconduction manner instead. At a higher bias voltage of *V*_DS_ = 2 V, the Schottky barrier at the InSe/NbTe_2_ interface vanishes and Ohmic contact with low contact resistance is accomplished;^25,29^ thus, the resistance of the whole device channel is determined by the resistance of InSe area on SiO_2_, as well as the contact resistance at Au–InSe interface results from MIGS.^30^ Consequently, considerable photocurrent is generated only when the laser beam illuminates the InSe area on SiO_2_, especially the area close to the “source” electrode, which is a common case for 2D phototransistors with uniform channel. These results indicate that the InSe/NbTe_2_ heterostructure device transitions from a vertical Schottky diode to a lateral phototransistor when the *V*_DS_ is increased from 0 V to a high forward bias of 2 V and works in “photovoltaic + photoconduction” hybrid mode when *V*_DS_ is close to *V*_OC_ under a certain illumination condition. In practical applications, the device could be configured to operate in photovoltaic or photoconduction mode, subject to the specific requirement (e.g., short response time, low dark current, high responsivity).^4^

## Conclusions

In conclusion, deterministic light-to-voltage conversion is accomplished in a two-dimensional diode, which incorporates a gate-independent Schottky junction and a gate-tunable homojunction. The rectification ratio and field effect current on/off ratio of this diode are close to and over 10^4^, respectively. The deliberate stacking sequence, where the semiconducting InSe is placed on metallic NbTe_2_, leads to so strong dielectric screening effect that the photovoltaic effect in this heterojunction is independent of gate voltage. This exceptional function is quite useful for light-to-voltage conversion in integrated photonic chips, and optical-to-electrical interconnects, as the output photovoltage is directly determined by the light input. In addition, the transition between photoconduction and photovoltaic effects in this device is revealed by scanning photocurrent mappings at various bias voltage *V*_DS_. In the future, the stable light-to-voltage conversion could be extended to infrared light with narrow-gap 2D semiconductors,^46,47^ and this device could be further optimized with other promising 2D metals with optional work function.^22,48^ The robust light-to-voltage conversion and flexible switching of operation mode demonstrated here are quite promising for the future on-chip optical-to-electrical conversion,^43,49^ as well as multimodal photodetection and analogue integrated electronics.

## Materials and Methods

### Device Fabrication

The device is fabricated with the same method employed in our recently published article.^37^ Thick flakes of NbTe_2_ and InSe are exfoliated from bulk materials and transferred to a Si wafer covered with 300 nm thick SiO_2_, assisted by polydimethylsiloxane (PDMS) stamps. The Ti/Au (5:100 nm) are patterned through electron beam lithography (EBPG 5000, Vistec Electron Beam, Germany), electron beam evaporation (MASA IM-9912), and lift-off in acetone. After annealing at 180 °C in high vacuum (∼10^–5^ mbar) for 2 h (AML-AWB wafer bonding machine), a 2 nm thick aluminum layer is deposited by electron beam evaporation, followed by atomic layer deposition (ALD, Beneq TFS-500) of 20 nm thick Al_2_O_3_. Finally, the second annealing process with identical conditions is conducted.

### Optoelectronic Characterization

The fabricated device is connected to a printed circuit board (PCB) by wire bonding, and the PCB is fixed on a translation stage accompanied by a scanning near-field optical microscope (SNOM) (WITec, Germany). Gate and drain-source voltages are applied with Keithley 2400 and 2401 systems, respectively. A customized LabVIEW program controls the translation stage and Keithley systems. Light illumination is provided with a 403 nm laser (Toptica Photonics, Germany), a 532 nm laser (WITec, Germany), or the built-in white light source of the SNOM system, through a 20× objective. All of the measurements are conducted in the air.

## Supporting Information Available

Optical microscope image of the device, Raman and photoluminescence characterization, AFM result, output *I*_DS_–*V*_DS_ curves under light illumination, and time-dependent photoresponse (PDF)
