# Supplementary material for: Deterministic Light-to-Voltage Conversion with a Tunable Two-Dimensional Diode
Source: ACS Photonics. 2022 Jul 21;9(8):2825–32. doi: 10.1021/acsphotonics.2c00727 (PMC9389648; doi:10.1021/acsphotonics.2c00727)
Supplement: Supplementary file 1 — ph2c00727_si_001.pdf [file ph2c00727_si_001.pdf]

Supporting Information for

Deterministic light-to-voltage conversion with a tunable two-dimensional diode

Mingde Du[1,2,*], Xiaoqi Cui[1,2], Bin Zhang[1,3], and Zhipei Sun[1,2,*]

[1]Department of Electronics and Nanoengineering, Aalto University, Espoo FI-02150, Finland

[2]QTF Centre of Excellence, Department of Applied Physics, Aalto University, Espoo FI-00076, Finland

[3]Key Laboratory of In-Fiber Integrated Optics of Ministry of Education, College of Physics and Optoelectronic Engineering, Harbin Engineering University, Harbin 150001, China

*E-mail: mingde.du@aalto.fi, zhipei.sun@aalto.fi.

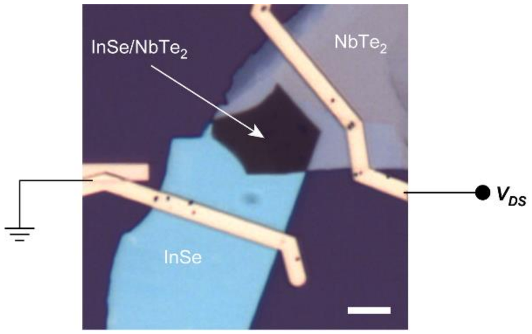

Figure S1. Optical microscope image of the InSe/$NbTe_2$ heterostructure device. The InSe and $NbTe_2$ flakes overlap at the dark area in the center. In the InSe/$NbTe_2$ overlapping area, the $NbTe_2$ flake is placed on the bottom and sandwiched between the SiO_2/Si substrate and the InSe flake. Scale bar, 5 μm.

Raman and photoluminescence characterization

Raman spectra of the two-dimensional materials are characterized by a WITec micro-Raman system, and the wavelength of excitation laser is 532 nm. Photoluminescence of semiconducting InSe flake is measured with SNOM system (WITec alpha300), the excitation laser has wavelength and power of 532 nm and 1 mW, respectively.

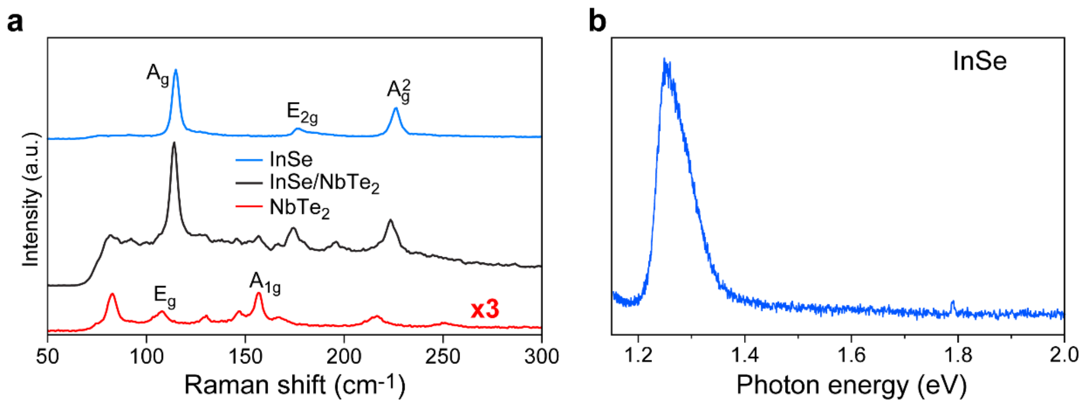

Figure S2. Raman and photoluminescence characterization of the 2D materials. (a) Raman spectra of the separate InSe and $NbTe_2$ flakes, as well as the InSe/$NbTe_2$ heterostructure. (b) Photoluminescence spectrum of the InSe flake.

The positions of major peaks in the Raman spectra of InSe (~115 cm^{-1}, ~177 cm^{-1}, ~227 cm^{-1}) and $NbTe_2$ (~83 cm^{-1}, ~108 cm^{-1}, ~157 cm^{-1}, ~217 cm^{-1}, ~252 cm^{-1}) are consistent with the previously published results.[1-4] The peak in the photoluminescence spectrum of InSe centered at ~1.25 eV, this value is in agreement with its bandgap that has been reported.[5]

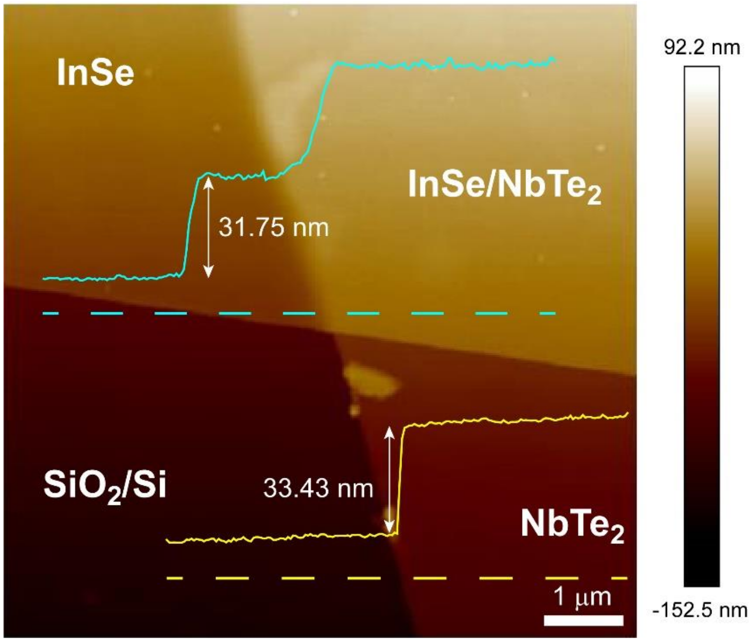

Figure S3. Atomic force microscope characterization of the 2D materials. The line profiles indicate that the thickness of InSe and $NbTe_2$ flakes are ~31.75 nm and ~33.43 nm, respectively.

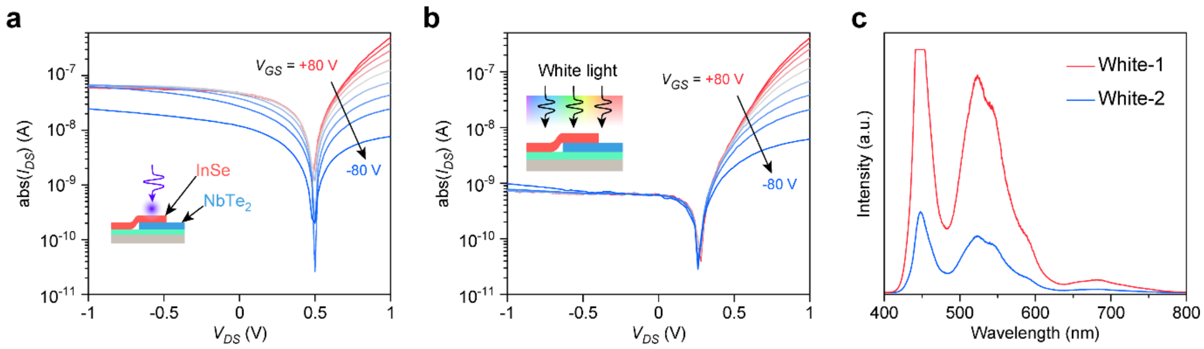

Figure S4. Output I_{DS}-V_{DS} curves measured under light illumination. (a) Output I_{DS}-V_{DS} curves under 403 nm laser illumination of 10 μW at the InSe/$NbTe_2$ heterostructure (inset). (b) Output I_{DS}-V_{DS} curves under white light illumination (inset). (c) The spectra of white light illuminations. The white light used in (b), the blue spectrum, has a much lower intensity than the white light (red spectrum) used in the measurement of Fig. 2c in the context.

All the results in Figure S4 and Figure 2(a, c) indicate that the V_{OC} generated in this device is independent of gate voltage.

Time-dependent photoresponse

In the measurement, the voltage between the source and drain electrodes of the heterostructure device is monitored with an oscilloscope, when a 520 nm laser is modulated with an optical chopper at 500 Hz (periodicity of 2 ms). The recorded signal (sampling rate = 1.25 MHz) is analyzed and processed with the "Signal Analyzer" App of MATLAB R2022a.

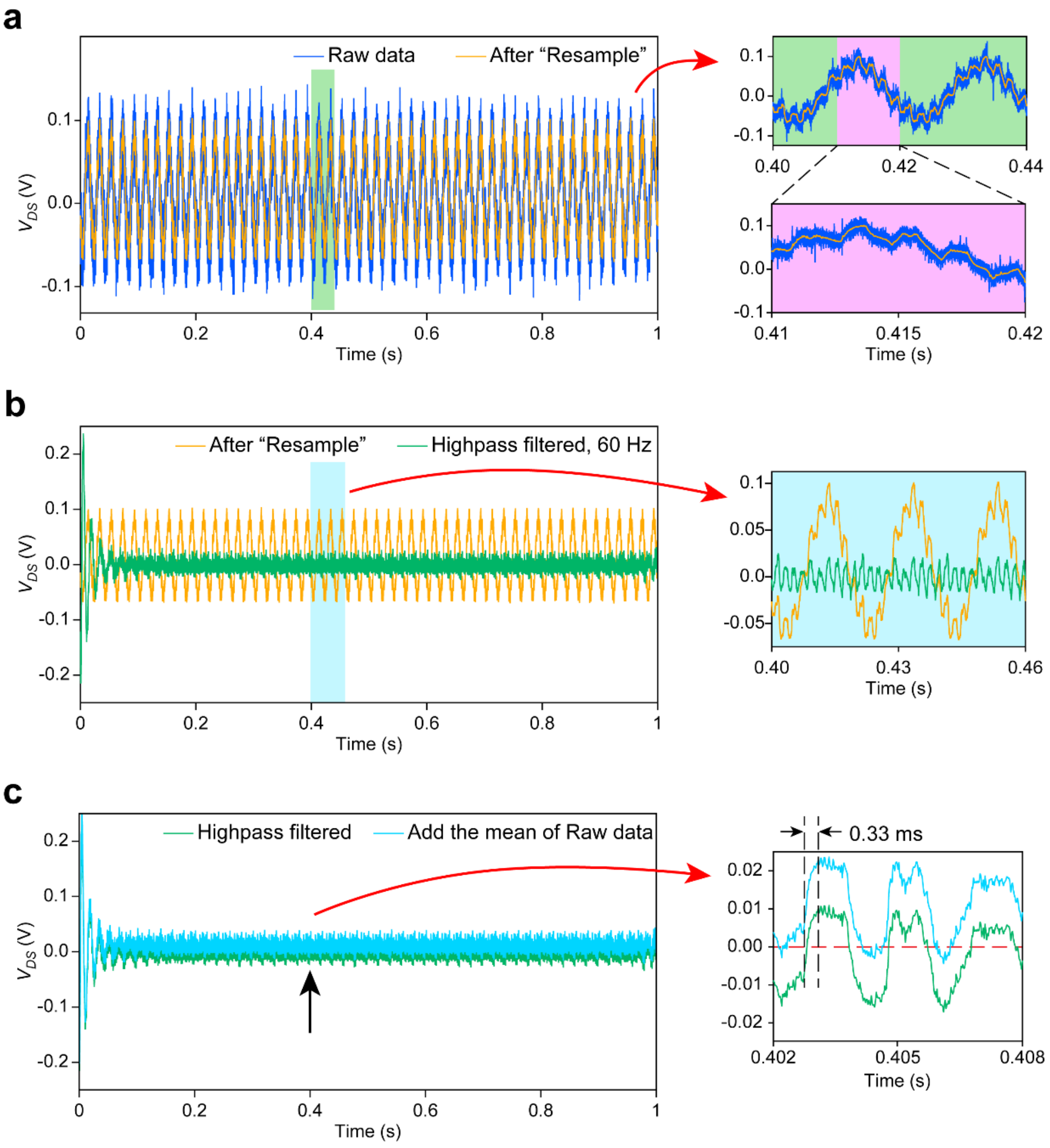

Figure S5. Time-dependent photoresponse. (a) The raw data with a 1.25 MHz sampling rate, and the processed data with a 50 kHz sampling rate obtained with "Resample" function in the "Signal Analyzer" App of MATLAB R2022a. (b) The signal obtained with "Resample" function, and a

“Highpass” filter with 60 Hz cut-off frequency. Apparently, the 50 Hz noise from the public AC power supply is effectively filtered, and the waveform of higher frequency is well preserved. (c) The highpass filtered signal, and result after adding the mean (0.0127 V) of the raw data in (a).

As indicated in the zoomed curve in Figure S5c, the AC voltage results from the photovoltaic effect induced by the modulated 520 nm laser have a periodicity of 0.002 second, which agrees with the periodicity obtained with the optical chopper (500 Hz, 2 ms). The response time of the heterostructure device working in photovoltaic mode is around 0.33 ms.

References

(1). Li, J.; Zhao, B.; Chen, P.; Wu, R.; Li, B.; Xia, Q.; Guo, G.; Luo, J.; Zang, K.; Zhang, Z.; Ma, H.; Sun, G.; Duan, X.; Duan, X. Synthesis of Ultrathin Metallic MTe_2 (M = V, Nb, Ta) Single-Crystalline Nanoplates. *Adv. Mater.* **2018**, 30, 1801043.

(2). Yan, Y.; Li, S.; Du, J.; Yang, H.; Wang, X.; Song, X.; Li, L.; Li, X.; Xia, C.; Liu, Y.; Li, J.; Wei, Z. Reversible Half Wave Rectifier Based on 2D InSe/GeSe Heterostructure with near-Broken Band Alignment. *Adv. Sci.* **2021**, 8, 1903252.

(3). Lei, S.; Ge, L.; Najmaei, S.; George, A.; Kappera, R.; Lou, J.; Chhowalla, M.; Yamaguchi, H.; Gupta, G.; Vajtai, R.; Mohite, A. D.; Ajayan, P. M. Evolution of the Electronic Band Structure and Efficient Photo-Detection in Atomic Layers of InSe. *ACS Nano* **2014**, 8, 1263-1272.

(4). Erdogan, H.; Kirby, R. D. Raman Spectrum and Lattice Dynamics of $NbTe_2$. *Solid State Commun.* **1989**, 70, 713-715.

(5). Bandurin, D. A.; Tyurnina, A. V.; Yu, G. L.; Mishchenko, A.; Zólyomi, V.; Morozov, S. V.; Kumar, R. K.; Gorbachev, R. V.; Kudrynskyi, Z. R.; Pezzini, S.; Kovalyuk, Z. D.; Zeitler, U.; Novoselov, K. S.; Patanè, A.; Eaves, L.; Grigorieva, I. V.; Fal'ko, V. I.; Geim, A. K.; Cao, Y. High Electron Mobility, Quantum Hall Effect and Anomalous Optical Response in Atomically Thin InSe. *Nat. Nanotechnol.* **2017**, 12, 223-227.
